# Role of bone marrow adipocytes in bone metastasis development and progression: a systematic review

**DOI:** 10.3389/fendo.2023.1207416

**Published:** 2023-08-29

**Authors:** F. Salamanna, D. Contartese, C. Errani, M. Sartori, V. Borsari, G. Giavaresi

**Affiliations:** ^1^ Surgical Sciences and Technologies, IRCCS Istituto Ortopedico Rizzoli, Bologna, Italy; ^2^ 3rd Orthopaedic and Traumatologic Clinic Prevalently Oncologic, IRCCS Istituto Ortopedico Rizzoli, Bologna, Italy

**Keywords:** bone marrow adipocytes, cancer cells, bone metastasis, bone microenvironment, systematic review

## Abstract

**Purpose:**

Bone marrow adipocytes (BMAs) are the most plentiful cells in the bone marrow and function as an endocrine organ by producing fatty acids, cytokines, and adipokines. Consequently, BMAs can interact with tumor cells, influencing both tumor growth and the onset and progression of bone metastasis. This review aims to systematically evaluate the role of BMAs in the development and progression of bone metastasis.

**Methods:**

A comprehensive search was conducted on PubMed, Web of Science, and Scopus electronic databases, following the Preferred Reporting Items for Systematic Reviews and Meta-Analyses (PRISMA) statement standards, to identify studies published from March 2013 to June 2023. Two independent reviewers assessed and screened the literature, extracted the data, and evaluated the quality of the studies. The body of evidence was evaluated and graded using the ROBINS-I tool for non-randomized studies of interventions and the Systematic Review Centre for Laboratory Animal Experimentation (SYRCLE) tool for *in vivo* studies. The results were synthesized using descriptive methods.

**Results:**

The search yielded a total of 463 studies, of which 17 studies were included in the final analysis, including 15 preclinical studies and two non-randomized clinical studies. Analysis of preclinical studies revealed that BMAs play a significant role in bone metastasis, particularly in prostate cancer followed by breast and malignant melanoma cancers. BMAs primarily influence cancer cells by inducing a glycolytic phenotype and releasing or upregulating soluble factors, chemokines, cytokines, adipokines, tumor-derived fatty acid-binding protein (FABP), and members of the nuclear receptor superfamily, such as chemokine (C-C motif) ligand 7 (CCL7), C-X-C Motif Chemokine Ligand (CXCL)1, CXCL2, interleukin (IL)-1β, IL-6, FABP4, and peroxisome proliferator-activated receptor γ (PPARγ). These factors also contribute to adipocyte lipolysis and regulate a pro-inflammatory phenotype in BMAs. However, the number of clinical studies is limited, and definitive conclusions cannot be drawn.

**Conclusion:**

The preclinical studies reviewed indicate that BMAs may play a crucial role in bone metastasis in prostate, breast, and malignant melanoma cancers. Nevertheless, further preclinical and clinical studies are needed to better understand the complex role and relationship between BMAs and cancer cells in the bone microenvironment. Targeting BMAs in combination with standard treatments holds promise as a potential therapeutic strategy for bone metastasis.

## 1 Introduction

**Figure 1:**
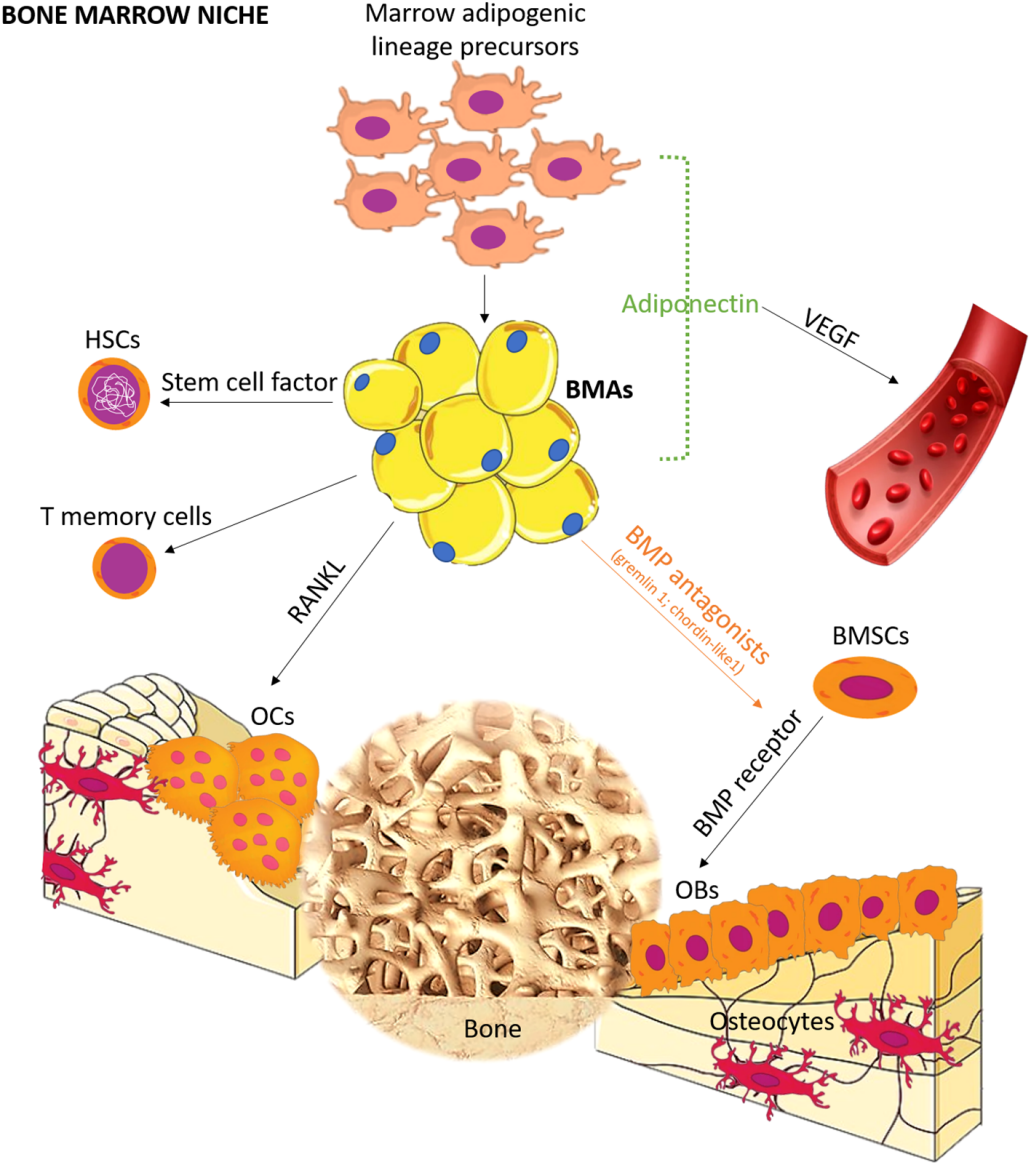
Schematic representation of the role of BMA lineage cell in bone marrow niche. BMAs form bone marrow niches that contain hematopoietic and bone cells. Distinct from canonical white, brown, and beige adipocytes, BMAs derived from bone marrow mesenchymal stromal cells possess unique characteristics and functions. Recent studies have revealed the differentiation pathway, and seminal works support the tenet that BMAs are critical regulators in hematopoiesis, osteogenesis, and osteoclastogenesis. OCs, osteoclasts; OBs, osteoblasts; BMSCs, bone marrow mesenchymal stromal cells; BMP, bone morphogenetic protein; HSCs, hematopoietic stem cells; RANKL, receptor activator of NF-κB ligand; VEGF, vascular endothelial growth factor.

Bone is a common site for distant metastasis, particularly in breast, prostate, and lung cancer (1). Once bone metastasis occurs, the prognosis is usually poor (1, 2). It can lead to skeletal-related events, such as pain, pathological fractures, compression of the spinal cord and nerves, and disruptions in calcium and phosphate homeostasis, significantly impacting patients’ quality of life (1). A retrospective analysis of electronic medical records indicated that prostate cancer patients had the highest risk of bone metastasis (2), with incidence rates of 18.0% at 1 year, 20.4% at 2 years, 24.5% at 5 years, and 29.2% at 10 years. Lung (10.4%–12.9%), renal (5.8%–9.9%), breast (3.4%–8.1%), gastrointestinal (2.3%–3.6%), malignant melanoma (1.6%–3.0%), and other tumors also presented varying rates of bone metastasis (2). The mechanism of bone metastasis remains elusive, although Paget’s “seeds and soil” hypothesis is commonly employed to explain this phenomenon (3). According to this hypothesis, tumor cells (seeds) find a favorable microenvironment (soil) in bone, enabling their survival, invasion, and growth (3). Over the years, several studies have focused on specific genes and cytokines (e.g., adhesive factors, inflammation factors, and chemotactic factors) associated with bone metastasis, with most of them prevalently related to “seed” rather than to “soil” (4). However, it is important to note that appropriate “soil” can affect the destiny of “seed”, as <0.01% of circulating tumor cells successfully develop distant metastasis (5). The bone marrow consists of various cell types, including red blood cells, white blood cells, platelets, endothelial cells, and bone marrow adipocytes (BMAs), all of which contribute to a “fertile soil” for tumor cells. Among these, BMAs are the most abundant cells in the bone marrow, and their prevalence increases with aging (6). In children, BMAs occupy ~15% of bone marrow volume, while in individuals aged 25 and above, they occupy ~70% (7). BMAs can be categorized into regulated BMAs (rBMAs) and constitutive BMAs (cBMAs) (8), with rBMAs found in the proximal part of bones representing red marrow and cBMAs found in the distal part comprising yellow marrow (8). These two types of BMAs differ in their development, gene expression, lipid content, and vascular density (8, 9). Although BMAs were traditionally considered passive “fillers” (6) in the bone marrow, recent studies have revealed their active role. BMAs are involved in different processes, such as bone remodeling, energy adaptation, and insulin metabolism by releasing adipokines, hormones, cytokines, and fatty acids (10–14). Importantly, the secretory profile of BMAs differs from that of adipocytes in other anatomical sites. For example, BMAs exhibit lower expression of leptin and adiponectin mRNA when compared to extramedullary adipocytes (15, 16), while they have higher tumor necrosis factor (TNF)-α and interleukin (IL)-6 (15). BMAs also present a pro-angiogenic and pro-apoptotic profile (17). In bone remodeling, BMAs release factors such as leptin and adiponectin, which stimulate the differentiation of multipotent stem cells into osteoblasts (**Figure 1**). However, BMAs can also release chemerin, an adipokine that inhibits osteoblastogenesis (12), and TNF-α and receptor activator of nuclear factor-κB-ligand (RANKL), which promote osteoclastogenesis (12). These functions highlight the critical role of BMAs in bone metabolism (12).

Based on this knowledge, recent research has focused on the potential relationship between BMAs and cancer bone metastasis. Studies have shown that BMAs can regulate the migration, invasiveness, and survival of tumor cells (18–21). These effects are mediated through various mechanisms, including the production and transfer of fatty acids (19) and the secretion of adipokines (22). Furthermore, tumor cells can create a microenvironment within the bone that supports their growth by exploiting the metabolic functions of BMAs. Notably, changes in BMAs induced by tumor cells involve a lipolytic state (19), increased expression of lipid transporter genes (19), and the induction of a senescence-associated secretory phenotype (23). Adipokines released by BMAs, such as adiponectin (24), leptin (25), adipsin (26), and estrogen (27), as well as pro-inflammatory cytokines like TNFα, IL-6, and RANKL (28, 29), have also implicated in bone metastasis. While these studies have increased our understanding of the molecular crosstalk between BMAs and cancer cells, the precise influence of BMAs on cancer onset and progression in bone is still not fully recognized. Therefore, this systematic review aims to summarize the research conducted in the last 10 years on the unique role of BMAs in supporting cancer bone metastasis.

## 2 Methods

### 2.1 Eligibility criteria

The PICOS model (population, intervention, comparison, outcomes, study design) was used to project this review: 1) studies that evaluated BMA function and role in bone metastasis in cells, animals, and patients (Population), submitted or not 2) to a specific intervention (Interventions), 3) with or without a comparison group (Comparisons), 4) that described BMA function and role in bone metastasis (Outcomes), in 5) preclinical (*in vitro* and *in vivo*) and clinical studies (Study design). Studies from March 22, 2013, to July 12, 2023, were included in this review if they met the PICOS criteria. The following were the exclusion criteria: studies evaluating 1) BMA function and/or role in primary cancer, 2) the role of mesenchymal stem cells (MSCs), 3) the efficacy of adipose-derived stem cells (ADSCs) as drugs for medical intervention, 4) mathematical modeling tool construction, 5) BMA variation in physiological conditions, 6) omental adipose-derived cells, 7) tumor progression in obesity, and 8) articles with partial data. Moreover, reviews, letters, comments to editor, meta-analysis, case reports, protocols and recommendations, editorials, guidelines, and articles not written in English were excluded.

### 2.2 Search strategies

The literature review involved a systematic search conducted in March 2023 according to the Preferred Reporting Items for Systematic Reviews and Meta-Analyses (PRISMA) statement (30). The search was conducted on three databases: PubMed, Scopus, and Web of Science. The resulting combination of terms was used ((bone metastases) AND (((adipose cells) OR (adipose tissue)) OR (adipocytes)), and for each of these terms, free words, and managed vocabulary specific to each bibliographic database were merged using the operator “OR”. The combination of free-vocabulary and/or Medical Subject Headings (MeSH) terms for the recognition of studies in PubMed, Scopus, and Web of Science are reported in **Table 1**.

**Table 1:** Combination of free-vocabulary and/or Medical Subject Headings (MeSH) terms for the identification of studies in PubMed, Scopus, and Web of Science.

**PubMed**	((“bone and bones”[MeSH Terms] OR (“bone”[All Fields] AND “bones”[All Fields]) OR “bone and bones”[All Fields] OR “bone”[All Fields]) AND (“metastasation”[All Fields] OR “metastasic”[All Fields] OR “metastasing”[All Fields] OR “metastasise”[All Fields] OR “metastasised”[All Fields] OR “metastasises”[All Fields] OR “metastasising”[All Fields] OR “metastasization”[All Fields] OR “metastasizes”[All Fields] OR “metastasizing”[All Fields] OR “neoplasm metastasis”[MeSH Terms] OR (“neoplasm”[All Fields] AND “metastasis”[All Fields]) OR “neoplasm metastasis”[All Fields] OR “metastase”[All Fields] OR “metastases”[All Fields] OR “metastasize”[All Fields] OR “metastasized”[All Fields]) AND (“adipocytes”[MeSH Terms] OR “adipocytes”[All Fields] OR (“adipose”[All Fields] AND “cells”[All Fields]) OR “adipose cells”[All Fields] OR (“adipose tissue”[MeSH Terms] OR (“adipose”[All Fields] AND “tissue”[All Fields]) OR “adipose tissue”[All Fields]) OR (“adipocytes”[MeSH Terms] OR “adipocytes”[All Fields] OR “adipocyte”[All Fields] OR “adipocytic”[All Fields]))) AND (2013:2023[pdat])
**Scopus**	(TITLE-ABS-KEY (bone AND metastases) AND TITLE-ABS-KEY (adipose AND tissue) OR TITLE-ABS-KEY (adipose AND cells) OR TITLE-ABS-KEY (adipocytes)) AND PUBYEAR > 2012 AND (LIMIT-TO (LANGUAGE, “English”)) AND (LIMIT-TO (DOCTYPE, “ar”))
**Web of Science**	(TS = bone metastases) AND (TS = adipose cells OR TS = adipose tissue OR TS = adipocytes)—with Publication Year from 2013 to 2023, English

### 2.3 Selection process

After the duplicate elimination by a public reference manager (Mendeley Desktop v.1.19.8), the potential pertinent articles were screened using the title and abstract by two reviewers (FS and DC). Studies that did not meet the inclusion criteria were eliminated, and any disagreement was resolved through debate until a consensus was reached or with the involvement of a third reviewer (GG). Finally, the remaining studies were comprised of the final stage of data extraction.

### 2.4 Data collection process and synthesis methods

The data extraction and synthesis started with cataloging the details of the studies. To increase validity and avoid omitting potential findings for the synthesis, two authors (FS and DC) extracted and made a table (**Supplementary Table 1**), taking into consideration the following: study type, primary tumor, experimental design, analyzed factors, BMAs’ role in bone metastasis, main results, and reference.

### 2.5 Risk of bias assessment

Two reviewers (FS and DC) individually analyzed the methodological quality of the included studies. In case of disagreement, they tried to reach a consensus; if this failed, a third reviewer (GG) made the definitive decision. The methodological quality of included clinical studies was evaluated by the ROBINS-I tool for the assessment of the risk of bias in non-randomized studies of interventions (48). The methodological quality of included *in vivo* studies was carried out according to the Systematic Review Centre for Laboratory Animal Experimentation (SYRCLE) tool (49), which has been originated to assess the risk of bias in animal studies. The risk of bias for *in vitro* studies was not assessed because, to our knowledge, for the type of *in vitro* studies included in this review, no standard quality assessment tool was present.

## 3 Results

### 3.1 Study selection and characteristics

**Figure 2:**
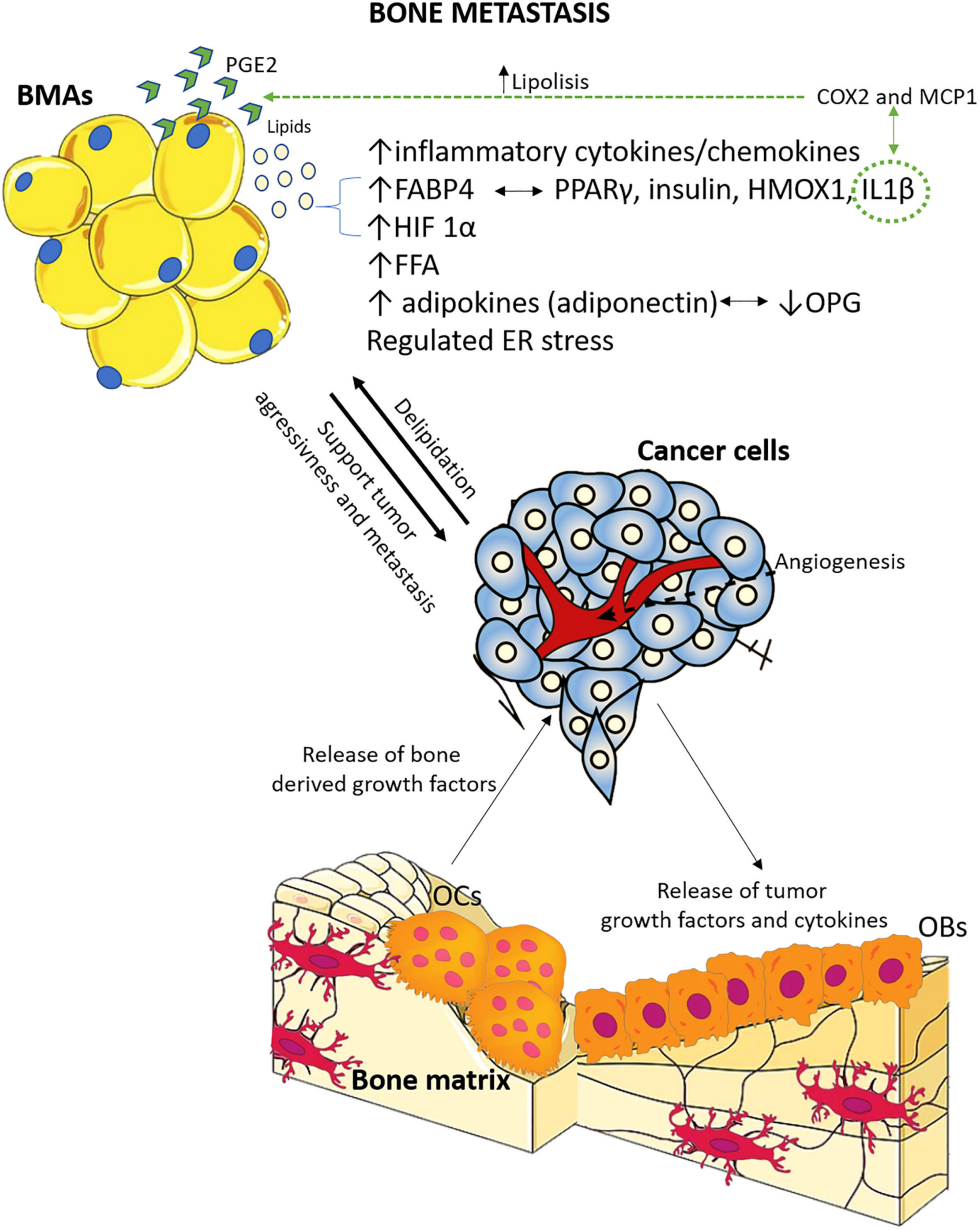
The PRISMA flow diagram for the systematic review detailing the database searches, the number of abstracts screened, and the full texts retrieved. PRISMA, Preferred Reporting Items for Systematic Reviews and Meta-Analyses.

The initial search found 484 studies. Of those, 110 were detected using PubMed, 217 using Scopus, and 157 using Web of Science. After screening the title and abstract, 53 articles were uploaded to Mendeley Desktop version 1.17.9 to remove duplicates. The resulting 26 complete articles were reviewed to determine whether the publication met the inclusion criteria, and 17 were considered eligible for the review. From the reference lists of the selected articles, no extra publications were found. Of the articles eligible for the review, two were non-randomized (retrospective) clinical studies, while the remaining 15 were preclinical studies, of which eight were both *in vitro* and *in vivo*, five only *in vitro*, and two only *in vivo*. The search strategy and study inclusion and exclusion criteria are specified in **Figure 2**.

**Figure 3:**
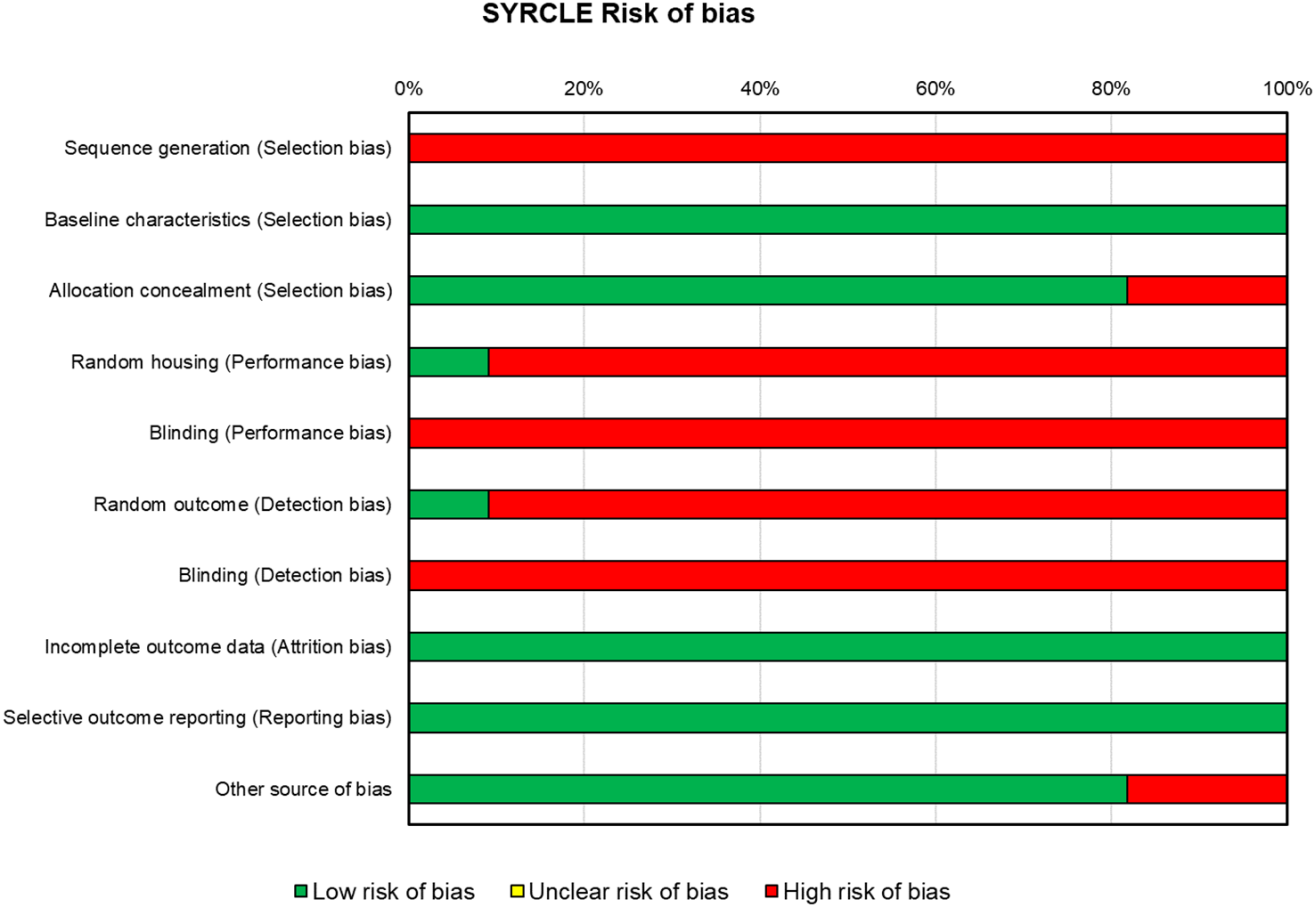
Schematic representation of the main factors and mechanisms potentially involved in the relationship between BMAs and bone metastasis, based on preclinical studies included in this systematic review. The relationship between tumor cells and bone cells is extensively studied, and it is known that tumor cells contribute to osteolytic bone disease, while resorbed bone releases factors that support tumor growth and survival. Adipocytes, located in proximity, play a crucial role in this interaction. There is evidence supporting both pro-tumor and anti-tumor effects of adipocytes, and there is also feedback from tumor cells to bone marrow adipocytes. OCs, osteoclasts; OBs, osteoblasts; ER, endoplasmic reticulum; OPG, osteoprotegerin; FFA, free fatty acids; HIF-1α, hypoxia inducible factor 1α; IL-1β, interleukin 1β; HMOX1, oxidative stress protein heme oxygenase 1; PPARγ, peroxisome proliferator-activated receptor γ; FABP4, fatty acid-binding protein 4; COX2, cyclooxygenase 2; MCP1, macrophage chemoattractant protein; PGE2, prostaglandin E2.

Of the preclinical studies, eight were on prostate cancer bone metastasis, one on breast cancer bone metastasis, one on melanoma bone metastasis, two both on breast and melanoma cancer bone metastasis, one on multiple myeloma bone metastasis, one on lung cancer bone metastasis, and one on specimens of different primary tumors bone metastasis from humans. Concerning the clinical studies, one was on breast cancer bone metastasis, while the other evaluated bone metastasis from different types of primary cancer. **Supplementary Table 1** describes the main characteristics of all the included studies. Furthermore, based on the preclinical studies’ main results, **Figure 3** summarizes the key factors and mechanisms potentially involved in the relationship between BMAs and bone metastasis.

### 3.2 Preclinical studies

#### 3.2.1 BMAs in prostate cancer bone metastasis

Eight studies have implicated BMAs as essential mediators of the development of prostate cancer bone metastases. In 2013, Herroon et al. (31) demonstrated in experimental models of intraosseous tumor growth and diet-induced obesity that marrow fat promotes the growth and progression of skeletal prostate tumors. Exposure to BMAs resulted in the induction of fatty acid-binding protein 4 (FABP4), controlled by fatty acids, peroxisome proliferator-activated receptor γ (PPARγ), insulin, interleukin 1 β (IL-1β), and heme oxygenase 1 (HMOX-1) in PC3 cells (human prostate cancer cells derived from bone metastasis). This stimulation of growth and invasiveness was also observed in obese mice with prostate cancer bone metastasis and in samples from prostate cancer patients with bone metastasis. The study highlighted the functional role of FABP4 in bone metastasis and the bi-directional interaction between FABP4 and PPARγ pathways (31). A subsequent study by the same research group (32) demonstrated that tumor-supplied IL-1β contributes to adipocyte lipolysis and regulates the pro-inflammatory phenotype in adipocytes by upregulating cyclooxygenase-2 (COX-2) and macrophage chemoattractant protein (MCP-1). This was associated with increased hypoxia signaling and activation of pro-survival pathways in PC3 cells. In another study, RNA sequencing analysis showed that exposure to adipocytes controls endoplasmic reticulum (ER) stress and the unfolded protein response signature in metastatic prostate cancer cells, partially through coordination by BIP/HSPA5, an ER chaperone (33). These findings were consistent with the overexpression of ER stress-associated genes in prostate cancer patients with bone metastasis. Diedrich et al. (34) demonstrated a functional relationship between BMA and cancer cells in bone *in vitro* and *in vivo* models of marrow adiposity. They showed that adipocyte-exposed cancer cells (PC3) exhibit increased expression of glycolytic enzymes, higher lactate production, and reduced mitochondrial oxidative phosphorylation, indicative of the Warburg phenotype. They also revealed that PC3 induces lipolysis of adipocytes as a potential maintenance mechanism. Additionally, adipocytes were found to drive the metabolic reprogramming of cancer cells through the oxygen-independent mechanism of hypoxia-inducible factor 1α (HIF-1α). Importantly, the metabolic signature observed in adipocyte-exposed cancer cells mimics the expression patterns observed in patients with metastatic bone disease. Another study demonstrated that soluble factors released by human primary BMAs can sustain the migration of prostate cancer cells in a CCR3-dependent manner, suggesting the potential involvement of the CCR3 pathway in prostate cancer cells homing to bone (35). This effect was enhanced by obesity and aging, which are known to increase the aggressiveness and the metastatic potential of prostate cancer cells. In humans, an improvement in CCR3 mRNA levels in prostate cancer bone metastatic samples *vs.* primary prostate cancer samples was also detected (35). These data were further confirmed by immunohistochemical evaluations that displayed overexpression of CCR3 in bone *vs.* visceral metastases. The study clearly underlined 1) the implication of the directed migration of CCR3 *in vitro* to the BMA production and 2) the enhancement of CCR3 that expresses cells in different human bone metastatic sites. These results suggested that the CCR3 pathway might be potentially implicated in prostate cancer cells homing to the bone. Wang et al. (36) highlighted the association between increased numbers of BMAs and the progression of prostate cancer cells in the bone marrow niche using *in vitro* and *in vivo* models. They demonstrated that a high-fat diet (HFD) in nude mice leads to dyslipidemia and specific alterations in the bone marrow, including increased adipocyte area and number, elevated level of free fatty acids (FFAs), and a decline in osteoblasts’ area and number. Furthermore, HFD stimulated COX2 expression and suppressed osteoprotegerin (OPG) expression in the bone marrow microenvironment. In a small cohort of patients, caprylic acid was identified as a specific FFA with higher levels in patients with prostate cancer bone metastases (n = 8) when compared to those without bone metastases (n = 8) or healthy controls (n = 16) (36). *In vivo* treatment of bone mesenchymal stem cells (BMSCs) with caprylic acid resulted in increased adipocyte differentiation and PPARγ expression, along with a subsequent reduction in osteoblast number. Treatment of BMAs with caprylic acid *in vitro* promoted BMSC-derived adipocyte differentiation, COX2 expression, PGE2 production, and antagonized osteoblastic differentiation and OPG release (36). These findings suggested that elevated caprylic acid levels may promote prostate cancer bone metastasis by stimulating adipogenesis over osteoblastogenesis. Higher FFA levels due to HFD may also influence prostate cancer bone metastasis by supporting a pro-tumor microenvironment through the increased numbers of BMAs (36). Additionally, utilizing an *in vivo* diet-induced models of BMA, Hardaway et al. (37), demonstrated a positive relationship between enhanced marrow fat content, bone degradation by ARCaP(M) and PC3 prostate cancer cells, and increased levels of host-derived CXCL1 and CXCL2, ligands of CXCR2 receptor. By also using an *in vitro* assay of osteoclastogenesis, it was shown that BMA conditioned media represented a significant source of CXCL1 and CXCL2 proteins and that both the conditioned media by adipocyte and the recombinant CXCL1 and CXCL2 ligands proficiently increase the maturation and differentiation of osteoclast (37). They further confirmed these data by stopping fat cell-induced osteoclast differentiation with CXCR2 antagonist or neutralizing antibodies. Finally, a recent study by Sanchis et al. (38), using a co-culture between PC3 and bone progenitor cells (MC3T3) or pre-osteoclastic cell line (Raw264.7), evaluated the PC3 cell transcriptome modulated by soluble factors secreted by bone precursors. By using transcriptomic data from human prostate cancer samples, the *in vitro* changes of metabolic genes allowed us to divide prostate cancer patients into two groups: primary prostate cancer and bone metastatic. Thus, the initial transcriptional profile activated the *in vitro* correlation with the clinical scenario. Furthermore, the expression levels of VDR, PPARA, SLC16A1, GPX1, and PAPSS2 metabolic genes resulted in independent risk-predictors of death in the SU2C-PCF dataset, and a risk score model constructed using this lipid-related signature can classify a subgroup of bone metastatic prostate cancer patients with a 23-fold higher risk of death. Comparing MDA-PCa-183 growing intrafemorally *vs.* subcutaneously in a patient-derived xenograft (PDX) model permitted us to authenticate this signature. Conditional media secretome analyses demonstrated that fibronectin and collagen type 1 were critical bone-secreted factors able to regulate tumor protein kinase A (PKA) (38).

#### 3.2.2 BMAs in breast and melanoma cancer bone metastases

Four studies have evaluated the interaction between BMAs and breast and melanoma cancer bone metastases. Gaculenko et al. (39) explored the influence of an HFD on tumor volume in an *in vivo* model of breast and melanoma cancer bone metastases. They initially characterized the effects of BMAs using the MDA-MB-231 breast cancer cell line in male immunocompromised RNU rats and established a malignant melanoma B16F10 model in male C57BL/6 mice. To translate their findings to patients, they used typical diagnostic techniques such as computed tomography (CT) and magnetic resonance imaging (MRI) combined with molecular analysis. The results demonstrated that breast and melanoma cancer bone metastases exhibited greater bone destruction in animals with high body weight, which is associated with a distinctive bone marrow environment characterized by enhanced bone marrow fat. In humans, BMAs were found to be co-localized with growing cancer cells. In addition, by analyzing the antagonization of PPARγ as a treatment for inhibiting bone tumor metastasis growth, they showed that targeting adipocytes through the inhibition of PPARγ, especially in overweight individuals, could reduce skeletal metastasis spreading and protect against cancer-associated bone loss. Similarly, Gregoric et al. (40) injected MDA-MB 231 breast cancer and B16F10 melanoma cells into the bones of nude rats or C57BL/6 mice treated with HFD and/or with the PPARγ antagonist bisphenol-A-diglycidyl ether (BADGE) to assess functional and metabolic parameters in the bone marrow. They characterized the pathophysiologic activities of bone metastases in obese and non-obese individuals. Using MRI, PET/CT, immunohistochemistry, and gene expression analysis, they highlighted that HFD in rats and mice resulted in enhanced tumor cell proliferation (Ki-67), glucose metabolism (LDHA, Gpi1, Slc16a3, and Angptl3), and angiogenic activity (CD31) in metastatic bone lesions. Wang et al. (42) demonstrated the importance of BMAs in bone metastasis using a bone metastasis mouse model obtained by an intracardiac injection of B16-F10 melanoma cells into immunocompetent C57BL/6 mice. They showed that the number of BMAs rapidly increased in the melanoma metastatic bone marrow niche. In addition, when co-cultured with melanoma cells *in vitro*, adipocytes were found to de-differentiate, and this process was dependent on the number of tumor cells (42). Delipidation of mature adipocytes was induced more at the highest density of melanoma cells than at lower densities. The expression of genetic markers of adipocytes, such as CCAAT/enhancer binding protein beta (CEBPβ), PPARγ, FABP4, and leptin, were decreased, while delta-like non-canonical Notch ligand 1 (Pref-1, IL-1β, IL-6, and C-C Motif chemokine ligand 2 (MCP-1)) was increased at the higher densities of melanoma cells. Using metastatic breast cancer cells, Templeton et al. (41) studied migration and colonization patterns of MDA-MB-231-fLuc-EGFP (luciferase-enhanced green fluorescence protein) and MCF-7-fLuc-EGFP breast cancer cells co-cultured with bone fragments isolated from 14 non-cancer patients. Breast cancer cell migration into tissues and toward tissue-conditioned medium was evaluated in Transwell migration chambers using bioluminescence imaging and analyzed in terms of secreted factors. Patterns of breast cancer cell colonization were also evaluated. The study observed higher migration of MDA-MB-231-fLuc-EGFP to bone in 12 samples compared to the control. These data were associated with a significant increase in adipokines/cytokines leptin and IL-1β. Fluorescence microscopy and immunohistochemistry of the bone fragments confirmed the colonization of breast cancer cells within the marrow adipose tissue compartment. The authors concluded that bone marrow adipose tissue and its molecular signals may play important roles as components of the breast cancer metastatic niche.

#### 3.2.3 BMAs in multiple myeloma bone metastasis

Regarding the interactions between BMA and myeloma cells, Liu et al. (43) demonstrated that BMA contributes to the persistence of myeloma-induced osteolytic lesions in patients in remission. This phenomenon is controlled by the secretion of adipokines that stimulate bone resorption and inhibit bone formation. Furthermore, they showed that myeloma cells can reprogram fat cells through PPARγ methylation, resulting in bone damage even after the successful elimination of myeloma. Adipocytes exhibit reduced PPARγ expression and a modified adipokine secretion profile after reprogramming by myeloma cells, leading to increased osteoclastogenesis and inhibition of osteoblastogenesis. This effect is regulated through the recruitment of specificity protein 1 (SP1) and polycomb repressive complex 2 (PRC2) proteins to the promoter region of PPARγ, inducing histone methylation of PPARγ at histone H3 lysine-27 trimethylation (H3K27me3) by the co-repressor complex.

#### 3.2.4 BMAs in lung cancer bone metastasis

In a recent preclinical *in vitro* and *in vivo* study, Luo et al. (44) investigated the effects of BMAs on bone metastases from lung cancer by comparing the mRNA expression level of bone metastatic SBC5 cells and non-bone metastatic SBC3 cells. The *in vitro* study revealed that the BMAs promote the invasion of bone metastatic SBC5 cells, but not non-bone metastatic SBC3 cells. SBC5 cells also stimulated the differentiation of osteoblasts and osteoclasts, as well as de-differentiation of mature BMAs. In an *in vivo* model of bone metastasis in NSG mice, rosiglitazone-induced bone marrow adiposity significantly enhanced SBC5-induced osteolytic lesion. RNA-seq analysis showed that S100A9 and S100A8 genes were upregulated in SBC5 cells compared to SBC3 cells. These findings suggest that the expression levels of S100A9/A8 may be critical for bone tropism in certain lung cancers. The elevated expression of S100A8/9 in SBC5 could play a role in the crosstalk between lung cancer cells and BMAs. Additionally, BMAs were found to significantly regulate the expression of the IL-6 receptor (IL6R), which is located adjacent to S100A8/A9 genes on chromosome 1q21.3.

#### 3.2.5 BMAs in different primary cancer bone metastases

In a recent study by Sato et al. (45), the role of BMAs on cancer cells was investigated in the context of bone metastasis from different primary sites (breast, kidney, lung, prostate, thyroid, uterus, colorectal, bladder, and others). The researchers compared the invasive fronts in bone metastasis with adipocyte-rich bone marrow (adipo-BM) to those with hematopoietic cell-rich bone marrow (hemato-BM). They found that the invasive front with adipo-BM had higher morphological complexity and an increased area of cancer-associated fibroblast (CAF) markers, suggesting a potential influence of adipocytes on cancer progression. Additionally, the adipo-BM invasive front exhibited a lower density of CD8+ lymphocytes and higher Ki-67 positivity, indicating increased cancer cell proliferation when compared to the tumor center. Interestingly, p21 positivity, a cell cycle regulatory protein, was significantly higher in cancer cells at the adipo-BM invasive front. The study also revealed that bone metastasized cancer cells acquired drug resistance-related gene expression profiles, suggesting a favorable tumor microenvironment provided by BMAs for cancer invasion and therapeutic resistance through CAF induction and immune evasion. These findings highlight the potential of targeting BMAs in the treatment of bone metastasis.

### 3.3 Clinical trials

Two retrospective clinical trials were found, one focused on breast cancer bone metastasis and the other on bone metastasis from various primary cancers including prostate, breast, lung, hepatoma, and rectal. In the first study, Goupille et al. (46) evaluated the fatty acid profile of breast adipose tissue specimens collected during surgery in 261 pre- and postmenopausal women with breast cancer to determine potential differences associated with bone metastases. The study highlighted that low levels of n-3 long-chain polyunsaturated fatty acids (n-3 LC-PUFAs) in breast adipose tissue were associated with the occurrence of bone metastases in premenopausal women. In the second study (47) involving 1,280 Taiwanese patients with bone metastases and varying levels of adiposity who underwent radiotherapy, Chuang et al. demonstrated that subcutaneous adipose tissue index (SATI) and visceral adipose tissue index (VATI) were independently correlated with bone metastasis.

### 3.4 Risk of bias assessment

**Figure 4:**
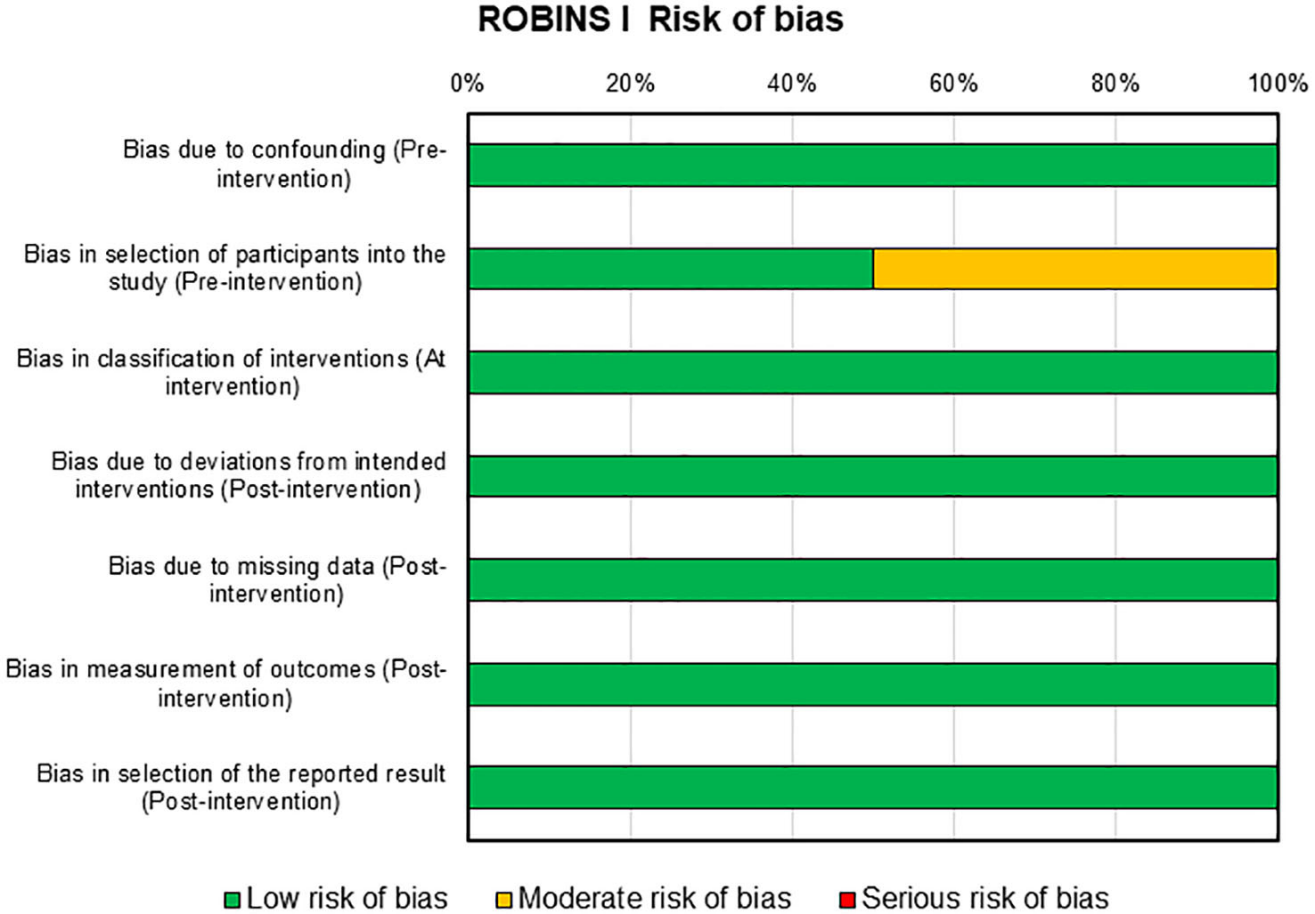
SYRCLE’s tool for assessing risk of bias in the *in vivo* studies. SYRCLE, Systematic Review Centre for Laboratory Animal Experimentation.

For the *in vivo* studies (n = 10), the risks of bias, reported in **Figure 4**, were high for almost all the papers. All included *in vivo* studies did not declare the method of sequence generation (n = 10). All the studies displayed that concerning baseline characteristics, groups were similar (n = 10). In approximately 80% of the studies (n = 8), the allocation was adequately concealed, while in two studies, it was not. One study (43) reported that animals were randomly housed during the experiment, while all other studies did not clearly report or did not use the housed blinding (n = 9). None of the studies selected assessor blinding, and only one study selected random outcome assessment. All studies included all the animals in the analyses (n = 10) and specified the primary outcomes (n = 10), and almost all were free of other biases that could result in high risk (n = 8).

**Figure 5:**
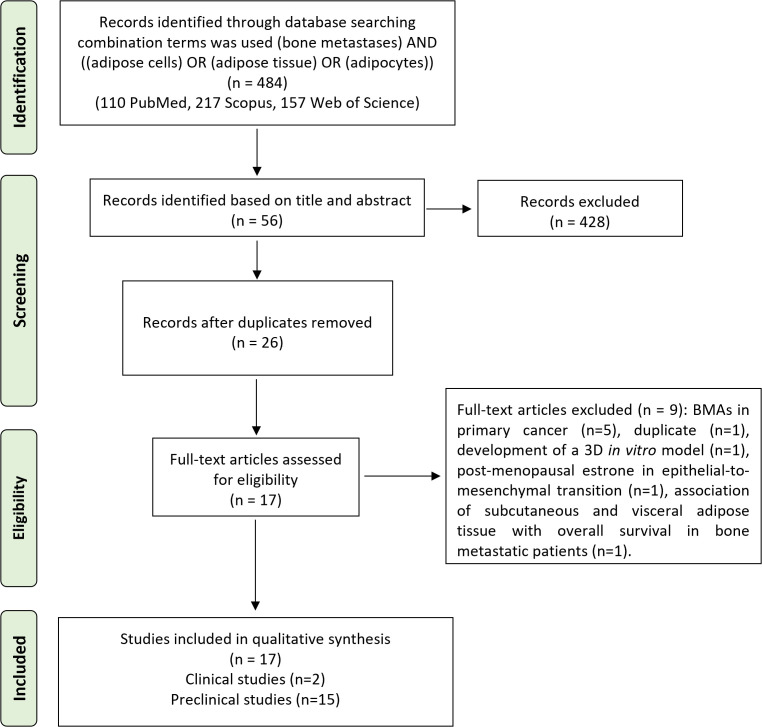
ROBINS-I tool for assessing risk of bias in non-randomized clinical studies.

Risks of bias assessment for the two clinical studies present in this review were reported in **Figure 5**. For these studies, the risk of bias was mainly low with only one domain in one study that presents a moderate risk, i.e., selection of participants into the study in pre-intervention (46).

## 4 Discussion

BMAs are unique in their origin and location, and they function as endocrine organs by releasing various products (13, 14), including adipokines, cytokines, chemokines, and growth factors. These products play a role in the promotion of bone metastasis in various types of cancer. This review highlights that BMAs are potential critical mediators of bone metastases in prostate, breast and, melanoma cancers. Adipocytes are the most abundant component in the bone metastatic microenvironment, and they have been shown to facilitate tumor cell recruitment, invasion, colonization, and proliferation (12–14). Most of the collected studies for this review are preclinical, and some of them have underlined the ability of BMAs to induce a glycolytic phenotype in metastatic prostate cells. This is achieved by increasing glycolytic enzymes and lactate production while decreasing mitochondrial oxidative phosphorylation (34, 40).

Glycolytic enzymes and proteins involved in glucose uptake are known to be regulated by HIF-1α (50–52). Under hypoxic conditions, HIF-1α can impair oxidative phosphorylation by promoting the expression of pyruvate dehydrogenase kinase and inhibiting the conversion of pyruvate to acetyl-CoA (53, 54). Consequently, highly glycolytic cells release lactate, which enhances tumor invasion and aggressiveness (55). Hypoxia also induces acidosis by increasing the acid load in the tumor microenvironment, leading to upregulated extracellular pH that further promotes cell proliferation in the acidic microenvironment (56). In addition to its effects on glycolysis, it has been observed that BMAs release soluble factors, such as CCL7, which directly promote the direct migration of metastatic prostate cancer cells to the bone in a CCR3-dependent manner. This effect is amplified by obesity and aging (35). The association between obesity and CCL7 levels is not surprising, as CCL7 is upregulated in several adipose tissue depots, and its serum levels are significantly higher in obese individuals compared to non-obese individuals (57, 58). Aging is also correlated with a senescent phenotype in mature adipocytes (59), which is associated with enhanced proinflammatory cytokine secretion, including CCL7, as well as metabolic disorders related to altered lipolysis and reduced glucose uptake (59, 60). In addition, CCL7 has been associated with the spread of colon and lung cancers to the liver (61, 62). Augmented levels of host-derived chemokines, CXCL1 and CXCL2, which bind to the G-protein coupled receptor CXCR2 (IL-8RB) expressed on macrophages, neutrophils, and epithelial cells, have been also reported. These chemokines showed a positive correlation with increased marrow fat and bone degradation during bone metastases (37). The link between these chemokines and inflammation in adipose tissue is critical for the metastatic bone tumor microenvironment (63–65).

In addition, it has been demonstrated that the enhanced growth and invasiveness of prostate cancer cells in the bone are affected by the upregulation of FABP4 and IL-1β by BMAs. FABP4 can contribute to cancer progression by providing a source of energy to cancer cells or by promoting angiogenesis, making it a critical factor in promoting invasive and infiltrative metastasis in bone. Similar findings have been observed in ovarian cancer as well (66–68). In contrast, IL-1β plays an important role in the inflammatory cascade by promoting the recruitment of inflammatory cells and increasing the production of other cytokines. These cytokines have a key effect on the progression of bone metastasis and many types of cancers (69). Multiple pieces of evidence have demonstrated that inflammation can stimulate bone metastasis through different mechanisms. Pro-inflammatory cytokines, immune factors such as chemokines and selectins, and other critical components of the tumor microenvironment play vital roles in tumor survival, proliferation, and metastasis (70–72). The microenvironment controls metastasis by sustaining the formation of a pre-metastatic niche, facilitating bone colonization, and regulating metastatic dormancy (70). This review also detected that interaction between prostate cancer cells and adipocytes leads to efficient crosstalk, which implicates not only tumor-supplied IL-1β but also the COX-2/MCP-1 pathways in adipocytes. These pathways contribute further to tumor aggressiveness and the reduction of osteoblast differentiation and physiological bone remodeling (32, 36). In addition to prostate cancer bone metastasis, preclinical studies on breast and melanoma cancer have also detected that BMAs affect skeletal tumor growth through increased osteoclast activity. This effect is mediated by the activator of nuclear factor-κB (RANK) and dendritic cell-specific transmembrane protein (DC-STAMP), a surface receptor required for osteoclast precursor fusion, as well as by IL-6 and PPARγ. The role of IL-6 in inflammation and tumor invasion is supported by its secretion by adipocytes (73–75). In contrast, PPARγ has a critical function in adipocyte differentiation and tumor growth due to its effect on the polarization of pro-tumorigenic macrophages (76, 77). PPARγ can affect different cell populations in the bone marrow, promoting the differentiation of bone marrow adipocytes while reducing the number of osteoblasts (78, 79). Upregulation of IL-6 and PPARγ by BMAs has also been observed in myeloma and lung bone metastatic diseases, leading to increased bone resorption and decreased bone formation (43, 44). However, there are limited data available for lung and myeloma bone metastatic diseases, probably because prostate and breast cancers have a “preferential attraction” for bone when compared to other cancer types.

Despite that much is still unknown about the connection between BMAs and bone metastasis, over the past decade, the contribution of BMAs to the establishment and progression of metastatic bone disease has become clearer. However, considering that aging and obesity result in increased numbers of BMAs, it is important to further understand the influence that these cells can have on the bone metastasis environment. Additionally, exploring potential gender-related differences in this context is also of key interest. To date, only two clinical retrospective studies have been conducted on BMAs and bone metastasis, and neither of these studies considered specific differences among patients in terms of aging, obesity, and gender. One of these studies evaluated a cohort of pre- and postmenopausal women with breast cancer, examining several clinical, biochemical, and histological factors related to bone metastases. This study found that reduced levels of long-chain n-3 polyunsaturated fatty acids in breast adipose tissue (LC-PUFA) were linked with bone metastasis development in premenopausal women.

The strength of this review lies in its systematic approach and comprehensive evaluation of studies retrieved from three different databases: PubMed, Scopus, and Web of Science. Furthermore, the review included all types of studies, including *in vitro*, *in vivo*, and clinical, systematically emphasizing the role of BMAs in the onset and progression of bone metastasis for the first time. Nevertheless, it is worth mentioning some limitations of this systematic review. The number of relevant articles included in the review is low, and some of the preclinical articles included had a high risk of bias in almost all the papers. Finally, inherent bias associated with the retrospective and descriptive nature of clinical study cannot be excluded.

Given the abundance of BMA in the bone marrow and their control over the formation and endocrine functions, targeting BMAs and their products could be a novel therapeutic strategy against metastatic bone disease. To date, therapeutic options for bone metastasis, such as bisphosphonates and monoclonal antibodies, although effective in reducing the prevalence of skeletal-related events in prostate and breast cancers, remain palliative. Based on the results of this review, it is evident that treatment strategies for bone metastases should target multiple components of the bone metastatic niche, including BMAs. Targeting BMAs in conjunction with standard treatments may provide a promising therapeutic alternative for bone metastasis in prostate and breast cancers. However, further preclinical and clinical studies are needed to better evaluate and understand the complex connections between BMAs and cancer cells in the bone microenvironment.

## Data availability statement

The original contributions presented in the study are included in the article/**Supplementary Material**. Further inquiries can be directed to the corresponding author.

## Author contributions

Conception and design of the work: FS. Acquisition of data for the work: FS and DC. Analysis of data for the work: FS, DC, MS, VB, and GG. Interpretation of data for the work: FS and DC. Drafting the work: FS and DC. Revising the work: FS, DC, MS, VB, CE, and GG. Approval for publication of the content: FS, DC, MS, VB, CE, and GG.
