# Supplementary material for: Role of bone marrow adipocytes in bone metastasis development and progression: a systematic review
Source: Front Endocrinol (Lausanne). 2023 Aug 29;14:1207416. doi: 10.3389/fendo.2023.1207416 (PMC10497772; doi:10.3389/fendo.2023.1207416)
Supplement: Supplementary file 1 [file Table_1.docx]

**Supplementary Table 1.** Main characteristics of preclinical studies and of non-randomized clinical studies included in the review.

| **Primary tumor** | **Study type** | **Experimental design** | **Analyzed factors** | **BMAs role in bone metastases** | **Main results** | **Ref.** |
| --- | --- | --- | --- | --- | --- | --- |
| ***Preclinical studies*** | | | | | | |
| Prostate | *In vitro* and *in vivo* | *In vitro*: BMSC isolated from femurs and tibiae of 6- to 8-week-old FVB/N mice to induce BMA differentiation. Culture of BMAs or adipocytes conditioned medium and PC3 cells  *In vivo*: HFD and PC3-DsRed cells intratibial injection in male mice FVB/N with mutation in Rag1 gene | FABP4, IL1β, PPARγ, HMOX-1 (histology, histomorphometry, immunohistochemistry, RT-PCR, immunofluorescence, western blot, ELISA) | Role of BMAs on growth and progression of skeletal prostate tumors *via* FABP4-dependent mechanisms | BMAs induces expression of lipid chaperone FABP4, IL1β, and oxidative stress protein HMOX-1 in metastatic tumor cells and stimulates growth and invasiveness. ↑ FABP4 in prostate skeletal tumors from obese mice and in bone metastasis samples from PCa patients. Bi-directional interaction between FABP4 and PPARγ | Herroon et al. 2013 (31) |
| Prostate | *In vitro* and *in vivo* | *In vitro*: co-culture of adipocytes and PCa cells (PC3 and ARCaP(M)) or conditioned media  *In vivo*: HFD, PC3 and ARCaP(M) cells intratibial and subcutaneous injection in 8- to 10-week-old male mice FVB/N with mutation in Rag-1 gene.  Bone biopsy tissue specimens obtained from PCa patients | IL1β, PGE2, COX2, MCP-1, mPGES, GLUT1, HIF-1α, VEGF, cyclin D, PPARD, IL1β/COX2/MCP-1 axis (histology, immunohistochemistry, RT-PCR, ELISA, lipidomic, western blot, immunocytochemistry) | Mechanisms driving a crosstalk between PCa cells and BMAs in the context of tumor survival | Metastatic PCa cells engage BMAs in a functional crosstalk that promotes IL-1β expression in tumor cells. Tumor-supplied IL-1β contributes to adipocyte lipolysis and regulates a pro-inflammatory phenotype in adipocytes via upregulation of COX-2 and MCP-1. ↑ activity of the IL-1β/COX-2/MCP-1 axis and ↑ in PGE2 production by adipocytes coincide with augmented hypoxia signaling and activation of pro-survival pathways in tumor cells | Herroon et al. 2019 (32) |
| Prostate | *In vitro* | BMSC isolated from tibiae and femurs of 6- to 8-week-old FVB/N mice to induce BMAS differentiation. Co-culture of BMA and PCa cells (PC3 or ARCaP(M)) | ER stress/UPR signature, BIP/HSPA5, ATF4, XBP1, SYVN1, DDIT3, HERPUD1, ASNS, E2F1, ATF6, CHAC1, TRIB3, DNAJB11, GDF15, PCK2, SESN2 (RNAseq, RT-PCR, western blot) | Impact of BMA on the transcriptome of bone metastatic PCa cells | Exposure to adipocytes drives ER stress/UPR signature in metastatic prostate tumor cells of different origin, coordinated at least in part by BIP/HSPA5. Several of the ER-stress associated genes overexpressed in metastatic samples from prostate cancer patients | Herroon et al. 2021 (33) |
| Prostate | *In* *vitro* and *in* *vivo* | *In vitro*: BMSC isolated from femurs and tibiae of 6- to 8-week-old FVB/N mice to induce BMAs differentiation; culture and co-culture of BMAs or adipocyte conditioned media and PC3 or ARCaP(M) cells  *In vivo*: HDF and PC3 or ARCaP(M) cells intratibial and subcutaneous injections in male mice FVB/N with mutation in Rag1 gene | ENO2, LDHa, PDK1, HK2, GLUT1, CS, IDH2, CA9, VEGF, *via* HIF-1α (RT-PCR, western blot, immunofluorescence, immunohistochemistry) | Modulation of tumor metabolism and adaptation within the bone microenvironment | BMAs promote glycolytic phenotype in metastatic PCa cells. In tumor cells exposed to adipocytes: ↑ expression of glycolytic enzymes, lactate production, ↓ mitochondrial oxidative phosphorylation. Adipocytes drive metabolic reprogramming of tumor cells *via* HIF-1α activation. The metabolic signature in tumor cells exposed to adipocytes mimics the expression patterns in patients with bone metastatic | Dietrich et al. 2016 (34) |
| Prostate | *In vitro* | BMAs and SAT obtained from male obese or elderly patients undergoing hip replacement surgery (age 51-73 years and BMI 20-25 kg/m^2^). Culture of PCa cells (C4-2B and PC-3) and BMA or SAT conditioned media | CCR3/CCL7 axis, CCR2, CCR1, CXCR1, CXCR2, CXCR4 (immunohistochemistry) | Implication of BMAs in homing of PCa cells to bone | Soluble factors released by BMAs (CCL7 and CCR3) support the directed migration of PCa cells in a CCR3-dependent manner. In human: enrichment of CCR3 in bone metastasis *vs.* primary tumors patients | Guérard et al. 2021 (35) |
| Prostate | *In vitro* and *in vivo* | *In vitro:* bone MSC-derived adipocytic differentiation; PC3/22RV1 cells and conditioned media obtained from adipocytes  *In vivo*: HFD in 8 4-week-old male nude mice (BALB/c-nu)  Human samples: normal individuals without PCa (n = 16), patients with PCa (n = 8), patients with PCa bone metastasis (n = 8) | COX2, OPG, PGE2, APN, CEBPα, PPAR-γ, RUNX2, MMP2, vimentin, EP2 (histology, immunohistochemistry, RT-PCR, western blot, ELISA) | Types of FFA involved in obesity related PCa bone metastasis | HFD ↑ area and number of adipocytes and ↓ area and number of osteoblasts; HFD promote COX2 and inhibit OPG expression in the bone. FFAs and caprylic acid ↑ in PCa patients with bone metastases. *In vitro*, caprylic acid promote bone MSC-derived adipocytic differentiation, COX2 expression, and PGE2 secretion and ↓ osteoblastic differentiation and OPG. Caprylic acid-treated adipocytes promote the invasion and migration of PCa cells and bone metastasis by dysregulated adipo-osteogenic balance of bone marrow | Wang et al. 2020 (36) |
| Prostate | *In vitro* and *in vivo* | *In vitro*: BMSC isolated from femurs and tibiae of 6- to 8-week-old FVB/N mice and induced to become BMAs. Co-culture of BMAs-conditioned media and PC3 or ARCaP(M) cells  *In vivo*: HFD, ARCaP(M) or PC3 cells intratibial injection in 8- to 10-week-old male mice FVB/N with mutations in Rag-1 gene | CXCL1, CXCL2, CXCR2, CALC, CAT K, MMP-9 (RT-PCR, ELISA, western blot) | Role of CXCL1/CXCL2 chemokines and their receptor CXCR2 in adiposity-induced osteoclastogenesis and prostate tumor-driven osteolysis of the bone | Positive correlation between increased marrow fat content, bone degradation by ARCaP(M) and PCa prostate tumors, and augmented levels of host derived CXCL1 and CXCL2, ligands of CXCR2 receptor. Media conditioned by BMAs is a significant source of CXCL1 and CXCL2 proteins. Adipocyte-conditioned media and the recombinant CXCL1 and CXCL2 ligands ↑ osteoclast maturation | Hardaway et al. 2015 (37) |
| Prostate | *In vitro* and *in vivo* | *In vitro*: Co-culture of PCa (PC3) and bone progenitor cells (MC3T3 or Raw264.7)  *In vivo*: MDA-PCa-183 PDX subcutaneously  or intrafemorally injection in 6- to 8-week-old male CB17 SCID mice  171 samples from primary prostatic (n = 14) or metastatic (n = 149) tumors from 63 PCa patients | VDR, PPARA, SLC16A1, GPX1 and PAPSS2, PKA signaling pathway, fibronectin, type-1 collagen (bioinformatics analysis, RNA-seq, RT-qPCR, secretome) | Metabolic gene alterations associated with the crosstalk between PCa and bone progenitor cells (pre-osteoblasts or pre-osteoclasts), and role of genes related to the metabolism of lipids in the survival of metastatic patients | VDR, PPARA, SLC16A1, GPX1 and PAPSS2 able to stratify PCa patients in primary PCa and bone metastatic. In PDX pre-clinical model, MDA-PCa-183 grow intrafemorally vs. subcutaneously, under the control of PKA | Sanchis et al. 2022 (38) |
| Breast and melanoma | *In vivo* | HFD, MDA-MB-231 cells intratibial injection in male 4-week-old immunocompromised RNU rats and B16F10 cells intratibial injection in male 6-week-old C57BL/6 mice  Bone metastasis specimens from women with breast cancer | RANK, DC-STAMP, col1a1, OCN, PPARγ, IL6, Ki67 (RT-PCR, CT, MRI, histology, immunohistochemistry) | Role of BMAs on skeletal tumor growth | HFD-induced BMAs lead to accelerated tumor progression and increased osteolytic lesions | Gaculenko et al. 2021 (39) |
| Breast and melanoma | *In vivo* | HFD, MDA-MB-231 cells intraarterially injection in 4–6 weeks old male RNU rats and B16F10 cells injection in C57BL/6 male 6-week-old mice | Functional and metabolic parameters in bone marrow, Ki67, LDHA, CD31 (MRI, PET/CT, histology, immunohistochemistry, immunofluorescence, RT-PCR) | Role of BMAs in the growth of disseminated tumor cells in the bone marrow | ↑ glucose metabolism and angiogenic activity in metastatic bone lesions in HFD-fed animals | Gregoric et al. 2022 (40) |
| Breast | *In vitro* | MDA-MB-231-fLuc-EGFP and MCF-7-fLuc-EGFP breast cancer cells in co-culture with cancellous bone tissue fragments isolated from 14 hip arthroplasties | Leptin, IL1β, OPN, OPG, IL6 (fluorescence histology, immunohistochemistry, BLI, secretome) | Parameters of human bone tissue associated with breast cancer cell osteotropism and colonization in the  metastatic niche | ↑ MDA-MB-231-fLuc-EGFP breast cancer cell migration to bone-conditioned vs. control medium in 12/14 specimens and ↑leptin and IL1β. | Templeton et al. 2015 (41) |
| Melanoma | *In vitro* and *in vivo* | *In vitro:* bone marrow MSC/murine  adipocyte cell line (14F1.1 cells) differentiated into BMAs; co-culture of BMAs/adipocyte conditioned media and B16-F10 cells  *In vivo*: intracardiac injection of B16-F10 murine melanoma cells in 10–12-week-old immunocompetent C57BL/6 male mice | FABP4, PPARγ, CEBPα, CEBPβ, leptin, APN, IGF1, IL6, IL1β, Pref-1, MCP-1 (histology, histomorphometry, RT-PCR, ELISA) | Behaviors of BMAs in metastatic niches during bone metastasis | ↑ number of BMAs in melanoma metastatic niches | Wang et al. 2017 (42) |
| Myeloma | *In vitro* and *in vivo* | *In vitro*: adipocytes co-cultured with patient derived CD138+ primary myeloma cells or human myeloma cell lines  *In vivo*: human bone chips subcutaneously implanted into NOD-*scid* IL2Rg^null^ mice and injection of conditioned medium of adipocytes isolated from patients with myeloma in remission or of adypocites exposed to human myeloma cells  Bone biopsy samples obtained from patients with myeloma in remission | PPARγ, PRC2, EZH2, SUZ12, APN, ADIPSIN, VISFATIN, TNFα, H3K27me3 (PCR, western blot, histology, immunohistochemistry, histomorphometry) | Role of BMAs in genesis of myeloma-associated bone disease | BMAs contribute to the persistence of myeloma-induced osteolytic lesions in patients in remission *via* secretion of adipokines that stimulate bone resorption and inhibit bone formation. Transformed plasma cells can reprogram adipocytes *via* increased methylation of PPARγ and altered adipokine production | Liu et al. 2019 (43) |
| Lung | *In vitro* and *in vivo* | *In vitro*: co-culture of bone metastatic SBC5 cells, non-bone metastatic SBC3 cells, and OP9 cells induced to mature BMAs  *In vivo*: Rosiglitazone-induced marrow adiposity and intra-femoral injection of SBC5 cells in B-NSG female mice (8–11 weeks old) | 1q21.3(S100A7/8/9‑IL6R) ‑TLR4 pathway, Pparg, MMP9, Il6, IL6R, S100A8, S100A9, PTHLH, Pth1r, Ager, Tlr4, Fabp4, Lep, Adipoq, Cxcl12, LEPR, ADIPOR1, ADIPOR2, CXCR4 (RNA-seq, RT-PCR, histology, microCT) | Effects of BMA on bone metastasis in lung cancer | BMAs ↑ invasion of SBC5 *in vitro*. SBC5 promote osteoblast and osteoclast differentiation and de-differentiation of mature BMAs. Rosiglitazone-induced marrow adiposity ↑ osteolytic lesion induced by SBC5 *in vivo*. IL6R was up regulated by BMAs. S100A8/A9 ↑ the osteoblastic differentiation and ↓ adipogenic differentiation, whereas TLR4 inhibitor TAK242 attenuated this effect | Luo et al. 2020 (44) |
| ***Clinical studies*** | | | | | | |
| Breast | Retrospective | 261 pre- and postmenopausal women with breast cancer | n-3 LC-PUFA (lipid analysis immunohistochemistry, histology, MRI, CT, PET) | Association between fatty acid of breast adipose tissue and occurrence of bone metastases | ↓ n-3 LC-PUFA in breast adipose tissue associated with the development of bone metastases in premenopausal women | Goupille et al. 2020 (46) |
| Lung, hepatoma, breast, prostate, rectal and others | Retrospective | 1280 Taiwanese patients (57.8% men and 42.2% women) with bone metastases with low- and high-adiposity undergo radiotherapy | SATI, VATI, muscle index (CT) | Association between clinical measures of adiposity and OS | Both SATI and VATI, but not muscle index, were associated with bone metastatic disease | Chuang et al. 2020 (47) |

**Abbreviations:** FFA: free fatty acid; HFD: high-fat diet; BMA: bone marrow adipocytes; PCa: prostate cancer; COX2: cyclooxygenase 2; OPG: osteoprotegerin; PGE2: prostaglandin E2; RT-PCR: Real-time polymerase chain reaction; APN: adiponectin; CEBP: CCAAT-enhancer–binding protein; PPAR-γ: peroxisome proliferator-activated receptor gamma; RUNX2: Runt-related transcription factor 2; MMP: matrix metallopeptidase; EP2: E receptor 2; MSC: mesenchymal stem cell; FABP4: fatty acid-binding protein 4; ELISA: Enzyme-Linked Immunosorbent Assay; IGF1: insulin-like growth factor-1; IL: interleukin; MCP-1: Monocyte chemoattractant protein-1; Pref-1: Preadipocyte factor 1; fLuc-EGFP: luciferase-enhanced green fluorescence protein; BLI: Bioluminescence imaging; OPN: osteopontin; PKA: Protein Kinase A; PDX: Patient-Derived Xenograft; IL6R: interleukin 6 receptor; PRC2: polycomb repressive complex 2; EZH2: enhancer of zeste homolog 2; SUZ12: suppressor of zeste 12 homolog; H3K27me3: histone H3 lysine-27 trimethylation; TNFα: Tumor necrosis factor alpha; ER: endoplasmic reticulum; UPR: unfolded protein response; BMSC: bone marrow stromal cells; VEGF: Vascular Endothelial Growth Factor; HMOX-1: heme oxygenase 1; CALC: calcineurin; CAT K: cathepsin K; SAT: subcutaneous adipose tissue; BMI: body mass index; MRI: magnetic resonance imaging; PET/CT: positron emission tomography/computed tomography; RANK: receptor activator of nuclear factor-κB; DC-STAMP: dendritic cell–specific transmembrane protein; col1a1: alpha-1 type I collagen; OCN: osteocalcin; HIF: hypoxia inducible factor; PDK: pyruvate dehydrogenase kinase; LDHa: lactate dehydrogenase; GLUT1: glucose transporter 1; HK2: hexokinase 2; ENO2: enolase 2; CA9: carbonic anhydrase 9; CS: citrate synthase; IDH2: isocitrate dehydrogenase 2; n-3 LC-PUFA: long-chain polyunsaturated fatty acids; SATI: subcutaneous adipose tissue index; VATI: visceral adipose tissue index; OS: overall survival.
